# Microbial elements as the initial triggers in the pathogenesis of polymorphic light eruption?

**DOI:** 10.1111/exd.13162

**Published:** 2016-11-29

**Authors:** VijayKumar Patra, Peter Wolf

**Affiliations:** ^1^ Center for Medical ResearchMedical University of GrazGrazAustria; ^2^ Research Unit for PhotodermatologyDepartment of DermatologyMedical University of GrazGrazAustria

**Keywords:** antimicrobial peptides, commensal/pathogen/damage‐associated pattern (CAMP/PAMP/DAMP), microbiome, polymorphic light eruption, vitamin D3 analogues

## Abstract

The primary trigger of polymorphic light eruption (PLE) remains to be uncovered. We hypothesize that PLE may be initiated by elements resulting from UV‐induced damage to microbial communities of the skin, leading to a cascade of events eventually resulting in the skin rash of the disease. One mechanism by which epidermal injury by UV radiation could trigger PLE are danger signals such as damage or pathogen associated molecular patterns DAMP/PAMPs or commensal‐associated molecular patterns (CAMPs). Such triggers could be produced due to UV‐induced stress on microbial communities of the skin and exacerbate inflammatory responses by inducing the innate immune system through antimicrobial peptides (AMPs) such as psoriasin, RNase7, HBD‐2 and LL‐37. These AMPs also actively take part in initiating adaptive immunity. That signals derived from microbial rather than human elements may initiate PLE is supported by series of observations, including the PLE‐protective effect of topically applied microbial‐derived DNA repair enzymes.

## 1 Background

As early as 1942, Epstein suggested a photoallergic concept for photodermatoses (S1). Since then, much progress has been made in understanding the pathophysiology of photodermatoses and their most common form, polymorphic light eruption (PLE)^1^ (S2–S13). However, specific photoantigens that initiate PLE have not yet been identified. We hypothesize that PLE may be initiated by elements resulting from UV‐induced damage to microbial communities of the skin, leading to a cascade of events eventually resulting in the skin rash of the disease.

## 2 Hypothesis and Premises

**Figure 1:**
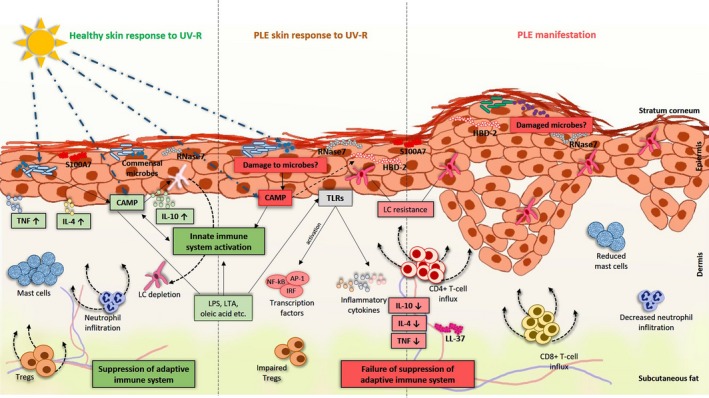
Hypothetical model for pathogenesis of PLE. Exposure of the skin to ultraviolet radiation (UV‐R) leads to the production of commensal‐associated pattern (CAMP). They are usually buried intracellularly, but upon secretion from dying human cells or microbial communities of the skin, they may exacerbate inflammatory responses by inducing the innate immune system through producing antimicrobial peptides (AMPs) such as S100A7 (psoriasin), HBD‐2, RNase7 and LL‐37. This increase in AMPs can promote in a vicious circle the activation of adaptive immune responses and exacerbate the inflammatory responses. In addition, UV‐R can also directly lead to microbial killing, resulting in the production of microbial signalling molecules such as lipopolysaccharides (LPS), lipoteichoic acid (LTA), oleic acid and others that in turn may lead to or enhanced abnormal immune responses through Toll‐like receptor (TLR) activation and transcription factors such as NF‐kB, AP‐1 and IRF. In healthy subjects, various cytokines such as TNF, IL‐4 and IL‐10 are expressed and infiltration of neutrophils and Tregs and increase in mast cell numbers in the skin are observed upon UV exposure. However, in PLE, there is a reduced production of these cytokines and decreased infiltration of neutrophils and reduced numbers of mast cells in the skin. Moreover, Langerhans cell resistance to UV‐R is seen in the skin of PLE patients compared to that of healthy controls. Taken together, all these events may be linked to the abrogation of UV‐R‐induced suppression of the adaptive immune responses in healthy subjects but a failure of suppression in PLE patients. Consequently, an influx of CD4+ T cells and CD8+ T cells is observed in the skin of PLE patients leading to inflammation and manifestation of the typical skin rash upon UV exposure

One mechanism by which UV‐R can trigger inflammation are potential danger signals, so‐called damage or pathogen‐associated molecular patterns DAMP/PAMPs (S16, S17). These DAMP/PAMPs may exacerbate inflammatory responses by inducing the innate immune system,^2^ for example by producing antimicrobial peptides (AMPs)^3^ (Fig. 1), and in turn, these AMPs could contribute to the induction of PLE. Similarly, damage to commensals may give rise to commensal‐associated molecular patterns (CAMPS) (S18, S19), resulting in microbial signals,^2^ altering the skin's microbial landscape and contributing to abnormal immune responses in inflammatory diseases such as psoriasis^4^ and beyond. In this regard, studies indicate that photoprovoked PLE patients show an upregulation of certain AMPs^5^ (S20). That signals derived from microbial rather than human elements may initiate PLE is consistent with a series of observations. First, the fact that PLE is a very common condition^6^ requires that the putative trigger should be ubiquitous. Undoubtedly, the skin's microbiome would fulfil this prerequisite. Second, PLE patients pretreated over 2 weeks twice daily with the topical vitamin D_3_ analogue calcipotriol showed reduction in PLE symptoms upon photoprovocation.^7^ Notably, it has been suggested that the beneficial effects of calcipotriol in inflammatory diseases are due to the modulation of AMP regulation via TH17 pathways.^5^ In addition, in vitro work in keratinocytes revealed that calcipotriol suppressed certain AMPs, which were stimulated by UVB.^8^ On the other hand, vitamin D_3_ has been found to upregulate LL‐37 in normal skin (S21) and narrowband UVB irradiation increased serum LL‐37 levels in psoriasis patients (S22). Indeed, direct effects of UV‐R could affect AMP expression 3 and contribute in starting the chain of events leading to PLE. Vitamin D is involved in modulating the gut microbial communities (S23), and downregulation of vitamin D receptor (VDR), associated with regulation of AMPs, is known to alter the microbial communities and their functions in murine intestine (S24, S25) and may have an antimicrobial effect also for skin (S26). Furthermore, low vitamin D levels were reported in patients with PLE, most likely due to sunlight avoidance (S11, S27, S28). Third, a lotion containing liposomal, unpurified microbial extracts with DNA repair capacity from *Anacystis nidulans* and *Micrococcus luteus* (a skin commensal) diminished PLE symptoms, without affecting the physiologic sunburn (erythema) response.^9^ The mechanism remained unclear; however, the direct effect of this enzyme approach on skin cells as measured by improved DNA repair was low (S29), and thus, we speculate that these extracts may have acted by repairing damage to commensals residing on or in the skin rather than reducing damage to human skin cells for which the approach has been intended for (S29, S30).

## 3 How to Test the Hypothesis

First of all, it needs to be ascertained that the microbiome of the skin does not differ between PLE patients and the healthy controls by sequencing microbial DNA isolated, for example using skin swabs (S31). Tape stripping can be used to isolate and subsequently quantify the skin's AMPs to study co‐relation between expression of AMPs and presence of microbial communities. Furthermore, high‐throughput sequencing of the adaptive T‐cell immune receptor repertoire can be used to determine the clonality of an infiltrate (S32, S33). In this context, an immunologic type IV reaction to a specific photoantigen should be accompanied by a monoclonal T‐cell infiltrate, whereas a reaction induced by multiple antigens and/or CAMPS/DAMPS/PAMPS should be not. To directly study the role of the microbiome in the initiation of PLE, experimental photoprovocation could be performed in patients with and without rigid skin disinfection (e.g. using topical chlorhexidine or povidone iodine) before repetitive UV exposure. Additionally, in vitro grown cultures of skin commensals obtained from PLE patients could be exposed to UV‐R and their extracts painted back onto human skin to investigate whether PLE can be provoked. Ideally, a mouse model of PLE would be desirable to study the mechanisms of the disease but the only such model currently available has limitations (S8). Finally, germ‐free mice, antibiotic‐treated or disinfected mice could be used to study the effects of UV‐R on the immune response by employing the contact hypersensitivity (CHS) model (S34). A microbiome of a certain quality and quantity could have a protective role against immune suppression and thus may contribute to reduction of UV‐induced immune suppression in PLE. For instance, downregulation of IL‐10 was one cytokine abnormality found in PLE (S2) and a recent study suggested a protective role of commensal microbiota‐derived IL‐10 in stroke and inflammatory disorders (S19). Moreover, peripheral blood mononuclear cells from healthy donors when cocultured with probiotics produced large amounts of IL‐10 (S35). Intriguingly, oral administration of a nutritional supplement containing lycopene, beta‐carotene and *Lactobacillus johnsonii* ameliorated UVA‐photoprovoked PLE (S36).

## 4 Relevance and Perspectives

If the role of the microbiome in PLE is tested and confirmed, this will not only unravel the pathogenesis behind PLE, possibly open up avenues to new and better therapeutic options, but also extend the knowledge to other areas such as the susceptibility to UV carcinogenesis. In this regard, a study by Lembo et al. (S37) and our previous work (S38) have suggested that susceptibility to PLE may be a protective factor in the formation of skin cancer. However, the question why PLE patients are resistant against UV‐induced immune suppression (S13–S15) still remains open but gender (S39) and genetic predisposition may mainly account for this resistance (S40–S42).

## Conflict of Interests

The authors have declared no conflicting interests.

## Supporting information

Data S1. Supplementary Information.
