# Supplementary material for: Microbial elements as the initial triggers in the pathogenesis of polymorphic light eruption?
Source: Exp Dermatol. 2016 Nov 29;25(12):999–1001. doi: 10.1111/exd.13162 (PMC5396279; doi:10.1111/exd.13162)
Supplement: Supplementary file 1 — Data S1. Supplementary Information. [file EXD-25-999-s001.docx]

**Supplementary References**

1. Epstein S. Studies in abnormal human sensitivity to light. IV. Photoallergic concept of prurigo aestivalis. J Invest Dermatol 1942: 5: 289-298.

2. Kolgen W, van Meurs M, Jongsma M, et al. Differential expression of cytokines in UV-B-exposed skin of patients with polymorphous light eruption: correlation with Langerhans cell migration and immunosuppression. Arch Dermatol 2004: 140: 295-302.

3. Wolf P, Gruber-Wackernagel A, Rinner B, et al. Phototherapeutic hardening modulates systemic cytokine levels in patients with polymorphic light eruption. Photochem Photobiol Sci 2013: 12: 166-173.

4. Gruber-Wackernagel A, Heinemann A, Konya V, et al. Photohardening restores the impaired neutrophil responsiveness to chemoattractants leukotriene B4 and formyl-methionyl-leucyl-phenylalanin in patients with polymorphic light eruption. Exp Dermatol 2011: 20: 473-476.

5. Schornagel I J, Sigurdsson V, Nijhuis E H, Bruijnzeel-Koomen C A, Knol E F. Decreased neutrophil skin infiltration after UVB exposure in patients with polymorphous light eruption. J Invest Dermatol 2004: 123: 202-206.

6. Janssens A S, Pavel S, Out-Luiting J J, Willemze R, de Gruijl F R. Normalized ultraviolet (UV) induction of Langerhans cell depletion and neutrophil infiltrates after artificial UVB hardening of patients with polymorphic light eruption. Br J Dermatol 2005: 152: 1268-1274.

7. Wolf P, Gruber-Wackernagel A, Bambach I, et al. Photohardening of polymorphic light eruption patients decreases baseline epidermal Langerhans cell density while increasing mast cell numbers in the papillary dermis. Exp Dermatol 2014: 23: 428-430.

8. Schweintzger N A, Bambach I, Reginato E, et al. Mast cells are required for phototolerance induction and scratching abatement. Exp Dermatol 2015: 24: 491-496.

9. Gambichler T, Terras S, Kampilafkos P, Kreuter A, Skrygan M. T regulatory cells and related immunoregulatory factors in polymorphic light eruption following ultraviolet A1 challenge. Br J Dermatol 2013: 169: 1288-1294.

10. Schweintzger N, Gruber-Wackernagel A, Reginato E, et al. Levels and function of regulatory T cells in patients with polymorphic light eruption: relation to photohardening. Br J Dermatol 2015: 173: 519-526.

11. Schweintzger N A, Gruber-Wackernagel A, Shirsath N, Quehenberger F, Obermayer-Pietsch B, Wolf P. Influence of the season on vitamin D levels and regulatory T cells in patients with polymorphic light eruption. Photochem Photobiol Sci 2016: 15: 440-446.

12. Kolgen W, Van Weelden H, Den Hengst S, et al. CD11b+ cells and ultraviolet-B-resistant CD1a+ cells in skin of patients with polymorphous light eruption. J Invest Dermatol 1999: 113: 4-10.

13. van de Pas C B, Kelly D A, Seed P T, Young A R, Hawk J L, Walker S L. Ultraviolet-radiation-induced erythema and suppression of contact hypersensitivity responses in patients with polymorphic light eruption. J Invest Dermatol 2004: 122: 295-299.

14. Palmer R A, Friedmann P S. Ultraviolet radiation causes less immunosuppression in patients with polymorphic light eruption than in controls. J Invest Dermatol 2004: 122: 291-294.

15. Koulu L M, Laihia J K, Peltoniemi H H, Jansen C T. UV-induced tolerance to a contact allergen is impaired in polymorphic light eruption. J Invest Dermatol 2010: 130: 2578-2582.

16. Harberts E, Gaspari A A. TLR signaling and DNA repair: are they associated? J Invest Dermatol 2013: 133: 296-302.

17. Jounai N, Kobiyama K, Takeshita F, Ishii K J. Recognition of damage-associated molecular patterns related to nucleic acids during inflammation and vaccination. Front Cell Infect Microbiol 2012: 2: 168.

18. Cario E, Brown D, McKee M, Lynch-Devaney K, Gerken G, Podolsky D K. Commensal-associated molecular patterns induce selective toll-like receptor-trafficking from apical membrane to cytoplasmic compartments in polarized intestinal epithelium. Am J Pathol 2002: 160: 165-173.

19. Benakis C, Brea D, Caballero S, et al. Commensal microbiota affects ischemic stroke outcome by regulating intestinal gammadelta T cells. Nat Med 2016: 22: 516-523

20. Patra V, Mayer G, Schmidbauer U, Gruber-Wackernagel A, Horn M, Wolf P. An insight into the expression patterns of antimicrobial peptides psoriasin, RNase7, HBD-2,-3 and LL37 in polymorphic light eruption. J Invest Dermatol 2015: 135: 79.

21. van der Does A M, Bergman P, Agerberth B, Lindbom L. Induction of the human cathelicidin LL-37 as a novel treatment against bacterial infections. J Leukoc Biol 2012: 92: 735-742.

22. Al-Mutairi N, Shaaban D. Effect of narrowband ultraviolet B therapy on serum vitamin D and cathelicidin (LL-37) in patients with chronic plaque psoriasis. J Cutan Med Surg 2014: 18: 43-48.

23. Bashir M, Prietl B, Tauschmann M, et al. Effects of high doses of vitamin D on mucosa-associated gut microbiome vary between regions of the human gastrointestinal tract. Eur J Nutr 2015.

24. Jin D, Wu S, Zhang Y G, et al. Lack of Vitamin D Receptor Causes Dysbiosis and Changes the Functions of the Murine Intestinal Microbiome. Clin Ther 2015: 37: 996-1009 e1007.

25. Wu S, Yoon S, Zhang Y G, et al. Vitamin D receptor pathway is required for probiotic protection in colitis. Am J Physiol Gastrointest Liver Physiol 2015: 309: G341-349.

26. Lucas R M, Gorman S, Geldenhuys S, Hart P H. Vitamin D and immunity. F1000Prime Rep 2014: 6: 118.

27. Gruber-Wackernagel A, Obermayer-Pietsch B, Byrne S N, Wolf P. Correction: Patients with polymorphic light eruption have decreased serum levels of 25-hydroxyvitamin-D(3) that increase upon 311 nm UVB photohardening. Photochem Photobiol Sci 2016: 15: 129.

28. Rhodes L E, Webb A R, Berry J L, et al. Sunlight exposure behaviour and vitamin D status in photosensitive patients: longitudinal comparative study with healthy individuals at U.K. latitude. Br J Dermatol 2014: 171: 1478-1486.

29. Wolf P, Maier H, Mullegger R R, et al. Topical treatment with liposomes containing T4 endonuclease V protects human skin in vivo from ultraviolet-induced upregulation of interleukin-10 and tumor necrosis factor-alpha. J Invest Dermatol 2000: 114: 149-156.

30. Yarosh D, Klein J, O'Connor A, Hawk J, Rafal E, Wolf P. Effect of topically applied T4 endonuclease V in liposomes on skin cancer in xeroderma pigmentosum: a randomised study. Xeroderma Pigmentosum Study Group. Lancet 2001: 357: 926-929.

31. Ganju P, Nagpal S, Mohammed M H, et al. Microbial community profiling shows dysbiosis in the lesional skin of Vitiligo subjects. Sci Rep 2016: 6: 18761.

32. Gazzola A, Mannu C, Rossi M, et al. The evolution of clonality testing in the diagnosis and monitoring of hematological malignancies. Ther Adv Hematol 2014: 5: 35-47.

33. Vieyra-Garcia P A M, Wei T, Gram Naym D, et al. STAT3/5 dependent IL-9 overexpression contributes to neoplastic cell survival in mycosis fungoides. Clin Cancer Res 2016: 22: 3328-3339.

34. Wolf P, Hoffmann C, Quehenberger F, Grinschgl S, Kerl H. Immune protection factors of chemical sunscreens measured in the local contact hypersensitivity model in humans. J Invest Dermatol 2003: 121: 1080-1087.

35. Niers L E, Timmerman H M, Rijkers G T, et al. Identification of strong interleukin-10 inducing lactic acid bacteria which down-regulate T helper type 2 cytokines. Clin Exp Allergy 2005: 35: 1481-1489.

36. Marini A, Jaenicke T, Grether-Beck S, et al. Prevention of polymorphic light eruption by oral administration of a nutritional supplement containing lycopene, beta-carotene, and Lactobacillus johnsonii: results from a randomized, placebo-controlled, double-blinded study. Photodermatol Photoimmunol Photomed 2014: 30: 189-194.

37. Lembo S, Fallon J, O'Kelly P, Murphy G M. Polymorphic light eruption and skin cancer prevalence: is one protective against the other? Br J Dermatol 2008: 159: 1342-1347.

38. Wolf P, Quehenberger F, Mullegger R, Stranz B, Kerl H. Phenotypic markers, sunlight-related factors and sunscreen use in patients with cutaneous melanoma: an Austrian case-control study. Melanoma Res 1998: 8: 370-378.

39. Damian D L, Patterson C R, Stapelberg M, Park J, Barnetson R S, Halliday G M. UV radiation-induced immunosuppression is greater in men and prevented by topical nicotinamide. J Invest Dermatol 2008: 128: 447-454.

40. Millard T P, Bataille V, Snieder H, Spector T D, McGregor J M. The heritability of polymorphic light eruption. J Invest Dermatol 2000: 115: 467-470.

41. McGregor J M, Grabczynska S, Vaughan R, Hawk J L, Lewis C M. Genetic modeling of abnormal photosensitivity in families with polymorphic light eruption and actinic prurigo. J Invest Dermatol 2000: 115: 471-476.

42. Millard T P, Fryer A A, McGregor J M. A protective effect of glutathione-S-transferase GSTP1*Val(105) against polymorphic light eruption. J Invest Dermatol 2008: 128: 1901-1905.
